# Fabrication of Cu Based Metallic Binder for Diamond Tools by Microwave Pressureless Sintering

**DOI:** 10.3390/ma11081453

**Published:** 2018-08-16

**Authors:** Shenghui Guo, Xiaolei Ye, Liang Wang, Sivasankar Koppala, Li Yang, Tu Hu, Jiyun Gao, Ming Hou, Longtao Hu

**Affiliations:** 1 State Key Laboratory of Complex Nonferrous Metal Resources Clean Utilization, Kunming University of Science and Technology, Kunming 650093, China; shguo78@hotmail.com (S.G.); Stoneye2@163.com (X.Y.); pepsiva9@gmail.com (S.K.); houmingkmust@163.com (M.H.); 2 State International Joint Research Center of Advanced Technology for Superhard Materials, Kunming University of Science and Technology, Kunming 650093, China; 3 National Local Joint Laboratory of Engineering Application of Microwave Energy and Equipment Technology, Kunming 650093, China; hutu1219@126.com; 4 Faculty of Metallurgical and Energy Engineering, Kunming University of Science and Technology, Kunming 650093, China; wangliangkmust@163.com (L.W.); hu921124hu@163.com (L.H.); 5 School of Chemistry and Environment, Yunnan Minzu University, Kunming 650093, China; zq1396835730@163.com

**Keywords:** microwave sintering, metallic binder, microstructure, mechanical properties

## Abstract

Microwave pressureless sintering (MPS) method is successfully applied in the fabrication of Cu based metallic matrix for diamond tools. The main purpose of this work is to obtain better mechanical properties when the metal binder of the diamond tools was prepared by the MPS method. The orthogonal experimental method is adopted to design the sintering process parameters. The optimized experimental conditions are suggested as 880 °C of sintering temperature, 375 MPa of cold pressure, and 35 min of withholding time. The contrastive investigation of the MPS and conventional pressureless sintering (CPS) are performed under optimized conditions. The microstructures information are obtained by scanning electron microscopy (SEM), X-ray diffraction (XRD), electron probe microanalysis (EPMA), and the necessary mechanical properties, such as relative density, hardness, and flexural strength are tested. Experimental results show that the MPS method, compared with CPS, can significantly improve the mechanical properties of the metallic matrix. The factors of relative density, hardness, and flexural strength increase 1.25%, 3.86%, and 6.28%, respectively. The possible sintering mechanism of the MPS method is also discussed. This work may provide a reference for the fabrication of metal-based diamond tools by microwave heating method.

## 1. Introduction

Metallic matrix is considered as a widespread binding agent for the diamond tool because of its excellent thermal conductivity, high hardness, and wears resistance [1]. Metal-based diamond cutting tools are widely used for sawing and grinding hard-brittle materials, such as stone, concrete, and ceramics [2]. Currently, the main strategies for manufacturing the metal matrix diamond tools include traditional hot pressure sintering (CHPS) and conventional pressureless sintering (CPS) [3,4]. For the CHPS method, the sintering process of metal-based diamond tools is usually divided into three stages [4]. Firstly, the diamond abrasives and matrix metallic powders are mixed as a raw material according to a predeter mined recipe. Next, the mixture is compacted by a capacity cold press, and the compaction pressure generally allows for 200 MPa to obtain the desired green compact. Finally, the green compact is loaded into a graphite mold for hot press sintering. Unlike the CHPS method, the graphite mold is not required in the third stage by using the CPS method. Simply put, the matrix metallic powders and the diamond abrasives are uniformly mixed and then cold pressed into a certain shape to perform CPS. In the above process, the sintering process is a crucial stage in determining the quality and costs of diamond tools. As for CHPS, it is possible to obtain better quality diamond tools and higher yield in a shorter period of time due to the effect of external pressure and graphite molds. However, a large amount consumption of graphite molds during the CHPS process is not conducive to resource conservation and environmental protection due to the used graphite becoming a solid waste pollutant [5,6]. When compared to the CHPS method, the CPS process avoids the consumption of high purity graphite, whereas so long sintering time and enough high sintering temperature should be adopted to promote the alloying reaction [5]. It is worth pointing out that for the CPS and CHPS methods, the energy absorption mode depends on the heat transfer and heat radiation pattern from outside to the inside of sample in the traditional resistance furnace, which is contrary to the direction of gas escape in the process of densification. Therefore, the contradictory direction will hinder gas evolution and microstructure shrinkage. In addition, the traditional sintering methods, including CHPS and CPS, will result in the consumption of excessive electric energy [7]. Therefore, it is a meaningful attempt to seek a novel sintering method with low-energy consumption and high-efficiency to manufacture metallic matrix diamond tools.

Since the 1950s, the application of microwave energy was paid greater attention in the field of material processing, such as the sintering of ceramics, etc. [8]. Especially, in recent years, microwave heating technology has been widely accepted due to its unique features, such as rapid heating, internal heating, and non-thermal effects [9]. Unlike the heating behavior of bulk metal in the microwave field, after Walkiewcz et al. reported moderate heating of metal powders ranging from Mg 120 °C to Fe 760 °C, a great number of researchers have studied microwave treated metal materials [9,10]. Breval et al., Mondal et al., and Anklekar prepared WC/Co alloy, 90W-7Ni-3Fe alloy, and PM copper steel by microwave sintering, respectively. It was found that microwave sintering resulted in mechanical properties than conventional sintering [11,12,13]. Chandrasekaran et al. investigated the melting behavior of metals, such as Pb, Sn, Al, and Cu in the microwave field. The results showed that the melting speed of microwave heating was twice as fast as the conventional method, and the energy efficiency was higher [14]. Reddy et al. studied the preparation of Al/Al_2_O_3_ composites by microwave sintering and achieved good progress [15]. Clean efficient microwave synthesis was highlighted in another study that metal phthalocyanines are synthesized [16].

Based on the analysis of the above references, we have reasons to believe that microwave energy can be used as an effective heating method for alloy preparation, metal smelting, or metal-based material preparation. Similarly, metal-based diamond tools can also be considered as metal-based/diamond composites. Therefore, we are eager to apply microwave sintering to the preparation of metal-based diamond tools. Our previous exploratory work studied microwave pressureless sintering (MPS) technology to manufacture FeCuCo-based diamond tool bits and microwave hot press sintering (MHPS) technology to prepare Fe-Cu-W-Sn-based diamond tools [4,17]. When compared to traditional pressureless sintering method, the microwave sintered sample exhibits more uniform microstructure, fewer hole defects, and better bonding strength of the diamond abrasive in the alloy matrix. While considering the advantages of microwave sintering and traditional sintering, previous experience provides a valuable reference.

This work is based on the previous stage of research and aims to broaden the application of microwave sintered metal-based diamond tools. Therefore, in this paper, the Cu based as a bonding metal matrix for diamond tools is successfully prepared by adopting single metal powders as a raw material via the MPS method. The orthogonal design was used to optimize the sintering conditions, and the diamond-containing metal matrix was prepared under optimized conditions by MPS and CPS methods, respectively. By referring to the principle of recipe design and the sintering schedule of such diamond tools, we propose a viable MPS sintering process. In addition, possible mechanisms were discussed to explain the superiority of the MPS method.

## 2. Experimental Method

### 2.1. Materials and Equipment

**Figure 1:**
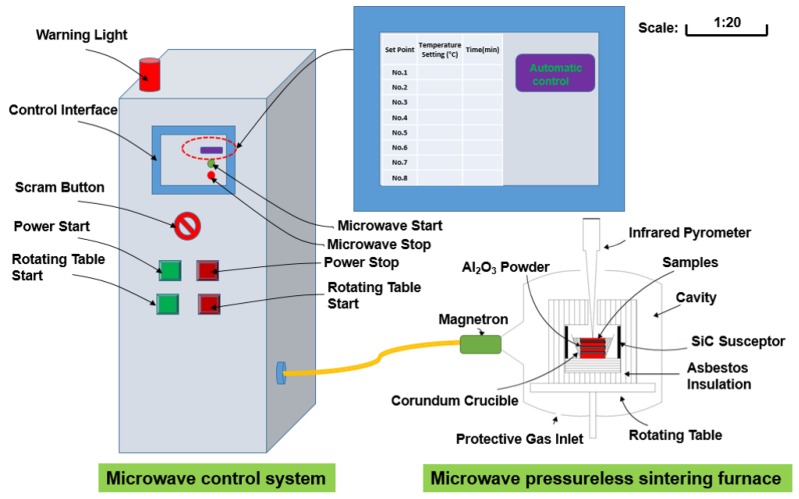
Schematic diagram of microwave pressureless sintering furnace and microwave control system.

Pure metallic powders (99.99%, 200 mesh) of copper (Cu), iron (Fe), Cobalt (Co), Tin (Sn), Nickel (Ni), and Titanium (Ti) were purchased from GRIPM Advanced Materials Co., Ltd. (Beijing, China), high purity diamond abrasive (99.98%, 35 mesh) was purchased from Henan Huanghe Whirlwind Co. Ltd. (Henan, China), and liquid paraffin was purchased from Sinopharm Chemical Reagent Co. Ltd. (Beijing, China). Three-dimensional vortex mixer (MX-2) and uniaxial automatic hydraulic press of 100T capacity (CP100) were purchased from Zhengzhou Golden Highway Co., Ltd., Zhengzhou, China. The experiments of the MPS process were carried out by home-made 6 kW microwave furnace with the frequency of 2.45 GHz (shown in Figure 1). As shown in Figure 1, the auxiliary heating of the SiC susceptor is added to avoid the weakening of the microwave effect due to the gradual densification of the sintered samples at the later stage of sintering, thereby affecting the sintering efficiency [8,18]. Firstly, the green compacts are placed in a corundum crucible in a rotating stage, and then protective gas is introduced. Secondly, after the microwave emitting device is activated, the rotating table is turned into uniformly applying the microwave to the sample. The temperature is detected by an infrared temperature measuring device and fed back to the microwave control system. It should be pointed out that the heating time can be controlled to achieve the purpose of controlling the heating rate. As a comparative test, CPS was performed by heating with resistance in a vacuum sintering furnace. In order to prevent the oxidation of the metal matrix at high sintering temperatures, each sintering experiment was performed in high pure Ar (99.99%) protective atmosphere.

### 2.2. Design of Sintering Schedule and Preparation of Samples

The metallic powders were mixed in a three-dimensional vortex mixer for 1 h to achieve uniform distribution blended starting powders. The mixed raw powders were compacted at a pressure of 200 MPa while using 100T capacity single-axis semi-automatic hydraulic press (CP100, Zhengzhou Golden Highway Co., Ltd., Zhengzhou, China) to make green compacts (30 mm × 12 mm × (6–6.5) mm). The detailed recipe of raw powder for metallic matrix was shown in Table 1. Then, the green compacts were sintered in MPS and CPS sintering furnace.

There is a fundamental difference between microwave heating and conventional heating. It is conceivable that microwave sintering is different from conventional sintering. At the same time, under pressureless conditions, the densification of the metal matrix relies on the diffusion filling of the liquid phase elements and the alloying process between the metal atoms. This may explain why the CPS method requires a slower heating rate and a longer sintering time. In addition, based on the previous studies [17], the higher heating rate and the shorter sintering time was allowed and no intermediate heat insulation process was required for the MPS method [4]. In this work, two main reasons will be clarified the choice of sintering temperature in the range of 820 to 900 °C. Firstly, pressureless sintering (MPS and CPS) was performed without external pressure, so higher temperature and longer sintering time were required to ensure the sufficient densification of the metal matrix. Secondly, the synthetic diamond abrasives had a distinct graphitization tendency when the sintering temperature was higher than 1000 °C. Therefore, choose relatively low temperature. The MPS process is as follows: From room temperature to 300 °C with the heating rate of 30 °C/min, and then heat up to 600 °C with 20 °C/min, finally up to the desired sintering temperature with 10 °C/min. Removing the sample from the furnace cooled to 180 °C after the incubation for 45 min. The entire process lasts about 160 min. Relatively, CPS schedule was set as follows: the room temperature was raised to 600 °C desired sintering temperature at the rate of 5 °C/min, then raised to the desired sintering temperature at the rate of 10 °C/min, the samples were incubated at 300 °C, 600 °C, and sintering temperature for 30 min, 30 min, 60 min, then removed the sample after the furnace cooled to 180 °C. The entire process lasts about 320 min. In this work, an *L*_9_(3^4^) orthogonal array was used to arrange experiments to investigate the effect of different sintering parameters (including sintering temperature, cold pressure, and holding time) on the mechanical properties of the metal matrix. 

### 2.3. Characterization and Measurements

One surface (12 mm × 6 mm) of the sintered sample was sequentially ground with 120#, 240#, 400#, 600#, 800#, and 1000# WC sandpaper. The polished sample is polished by a metallographic polishing machine (PG-1, Shanghai Xiguang Industrial Co., Ltd., Shanghai, China) until the surface is scratch-free and mirror-finished. Scanning electron microscopy (SEM, JSM-5610LV, JEOL, Ltd., Tokyo, Japan), X-ray diffraction (XRD-7000S, Shimadzu, Kyoto, Japan), and electron probe microanalysis (EPMA, JXA-8230, JEOL, Ltd., Tokyo, Japan) methods were used to obtain the microstructure information of the samples, which were helpful for the deter mination of corresponding relation between different sintering conditions and mechanical properties of sintered samples. Archimedes submerging method was used to measure the density of the sintered samples and then calculate the relative density. The HR-150A hardness tester was used to check the hardness Rockwell B of the samples. The hydraulic universal testing machine (AG-10TA) was used to test flexural strength of the sintered samples (The standard for bending tests is GB/T5319-2002, the values used for span and head speed are 25 mm and 1.0 mm/min, standard specimens with the dimension of 30 mm × 12 mm × 6 mm). The flexural strength calculation method, as follows [19]:

(1) $$\;R_{tr}=\frac{3fL}{2bh^{2}}\;\;$$

where *R_tr_* is a flexural strength (MPa); *f* is the force required for fracture (N); *L* is the span (mm); and, *b* and *h* are the width and thickness of the sample, respectively. At the same time, in order to study the holding force of the metal matrix on the diamond, the concept of the flexural strength reduction rate *q* and the holding force coefficient *F* were introduced as:

(2) $$\;q=\frac{\delta -\delta_{D}}{\delta }\;\;$$

(3)  F=100%−q  

where, *q* is the flexural strength reduction rate (%); *δ* is the flexural strength of the metal matrix (MPa); *δ_D_* is the flexural strength of diamond-containing metal matrix (MPa); and, *F* is the holding force coefficient.

## 3. Results and Discussion

### 3.1. Orthogonal Experimental Analysis

**Figure 2:**
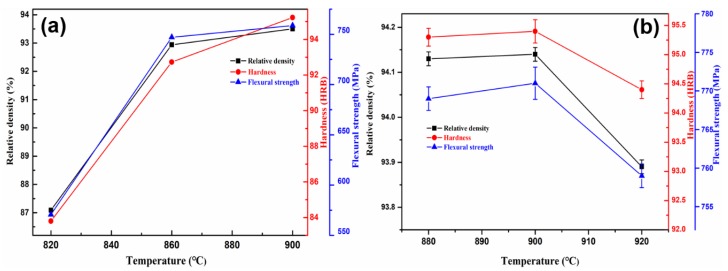
The effect of sintering temperature on the mechanical properties of the samples ((**a**) Orthogonal experimental; and (**b**) Conditional experiment (375 MPa, 35 min)).

**Figure 3:**
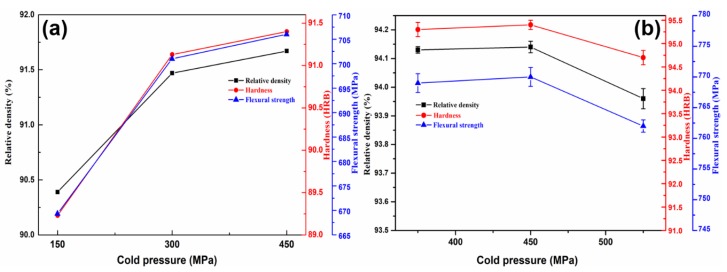
The effect of cold pressure on the mechanical properties of the samples ((**a**) Orthogonal experimental; and (**b**) Conditional experiment (880 °C, 35 min)).

**Figure 4:**
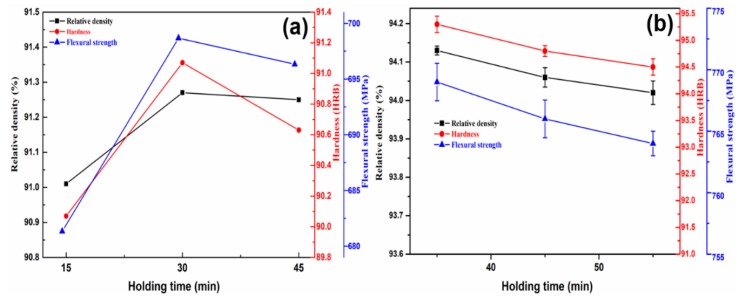
The effect of holding time on the mechanical properties of the samples ((**a**) Orthogonal experimental; and (**b**) Conditional experiment (880 °C, 375 MPa)).

**Figure 5:**
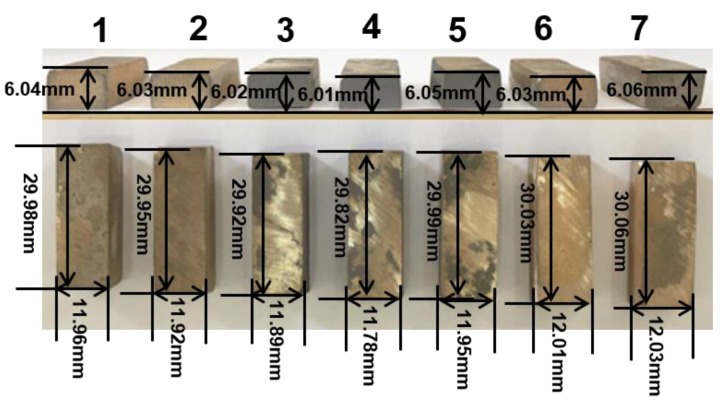
The images of microwave pressureless sintered samples: the sintering conditions of samples Nos. 3, 4 and 5 were 880 °C/375 MPa/35 min, 900 °C/375 MPa/35 min, and 920 °C/375 MPa/35 min, respectively; the sintering conditions of samples Nos. 3, 2, and 1 were 880 °C/375 MPa/35 min, 880 °C/450 MPa/35 min, and 880 °C/525 MPa/35 min, respectively; the sintering conditions of samples Nos. 3, 6, and 7 were 880 °C/375 MPa/35 min, 900 °C/375 MPa/45 min, and 920 °C/375 MPa/55 min, respectively.

It is well known that the orthogonal design is a commonly used experimental design method. In this work, an orthogonal design was used to arrange experiments to study the effect of different MPS conditions on the sintering effect (Table 2). The experimental results are showed in Table 3 and Table 4 and Figure 2a, Figure 3a, Figure 4a and Figure 5a. The rightmost three columns of Table 2 are the value of the evaluation index, i.e., relative density, hardness, and flexural strength of the sintered samples. Through the range analysis of Table 2 data, Table 3 was obtained. Table 3 was the range analysis of three evaluation indexes. In Table 4, *X_et_*, *Y_et_,* and *Z_et_* is the sum of the relative density, hardness, and flexural strength of all orthogonal experimental results. *X_et_*/3, *Y_et_*/3, and *Z_et_*/3 are the average value of the *X_et_*, *Y_et_,* and *Z_et_*, with the meaning of the evaluation indexes value for level *i* under factor *e* (*e* = A, B, C; *t* = 1, 2, 3). When the factor is certain, the difference in these average values of *X_et_*, *Y_et_,* and *Z_et_* is indicated influence degree of the levels for factor *e* (*e* = A, B, C; *t* = 1, 2, 3) on the relative density, hardness, and flexural strength of all orthogonal experimental results. Through range analysis of Equations (4)–(6), the range value *R_X_*, *R_Y_,* and *R_Z_* of *X_et_*, *Y_et_,* and *Z_et_* are obtained, as follows [20]:

(4) $$\;R_{X}=max\left(\frac{X_{et}}{3}\right)-min\left(\frac{X_{et}}{3}\right)\;\left(e=A,B,C;t=1,2,3\right)\;\;$$

(5) $$\;R_{Y}=max\left(\frac{Y_{et}}{3}\right)-min\left(\frac{Y_{et}}{3}\right)\;\left(e=A,B,C;t=1,2,3\right)\;\;$$

(6) $$\;R_{Z}=max\left(\frac{Z_{et}}{3}\right)-min\left(\frac{Z_{et}}{3}\right)\;\left(e=A,B,C;t=1,2,3\right)\;\;$$

The effects of the three factors, namely, *A*, *B*, *C* on the relative density, hardness, and flexural strength based on the range analysis are shown in Figure 2, Figure 3 and Figure 4.

It can be seen from Table 3 that the sintering temperature, the cold pressure, and holding time have the same influence trends on the density, hardness, and flexural strength of the sintered samples. In addition, the sintering temperature has a most obvious effect on the density, hardness, and flexural strength of the sintered sample and cold pressure is second, holding time has the minimal effect. Therefore, the sintering temperature is considered to be important factor affecting the performance of the sintered samples. Detailed analysis of the influence between mechanism performance and three sintering parameters are as follows.

Firstly, the sintering temperature mainly enhances the mechanical properties of sintered samples by promoting the formation of liquid phases and internal alloying reactions in the sintered samples. It can be clearly seen in Figure 2a and Table 3 that the relative density, hardness, and flexural strength of the sintered Cu-based samples increase with the increase of sintering temperature. There may be two reasons account for this phenomena. On the one hand, low-melting Sn is a liquid phase element, melts into the gap between other particles under the action of the capillary force to form a bonding medium [4]. On the other hand, it is an alloying reaction between elements, such as Cu and Sn, diffuses into each other to form an alloy [18]. As the temperature increases, the amount of liquid phase gradually increases, and the pores in the matrix are continuously filled so that the porosity gradually decreases, and the mutual diffusion of copper and tin intensifies. Therefore, the mechanical properties of sintered samples are also gradually improved. It is further observed that the increasing tendency of mechanical properties is obvious in the sintering temperature range from 820 °C to 860 °C, namely the relative density, hardness, and flexural strength increased by 6.7%, 10.7%, and 23.6%, respectively. At the same time, a noteworthy phenomenon displays that the mechanical properties only increased by 0.6%, 2.7%, and 1.5%, respectively, once the temperature increased from 860 to 900 °C. The following reasons can be responsible for this changing trend. In the low sintering temperatures stage, only a little liquid phase and the low activity of the metallic powder particles are inadequate to trigger sufficient alloying reaction, resulting in an undesirable relative density and hardness of the sintered samples. Due to low flexural strength when the samples were sintered at low temperature, it may ascribe to the incomplete grain development because of slow grain growth and movement rate, in turn, the crack easily spreads along grain boundary in the measurement of fracture [21]. As such, when the sintering temperature is moderately raised after the low-temperature stage, it can significantly increase the amount of liquid phase, enhance the alloying reaction, accelerate the migration rate of metallic elements, and finally improve the mechanical properties of sintered samples. However, excessive high sintering temperature leads to grain coarsening and a high grain boundary movement rate. When the motion rate of the grain boundary is faster than the rate of gaseous emission, it means that some pores will be surrounded by adjacent grains and resulting in a decline of hardness, relative density, and flexural strength. In addition, excessive sintering temperatures, especially exceeding 900 °C, will also give rise to the graphitization transformation of diamond phase. Therefore, it is critical to choose a suitable sintering temperature since both sintering efficiency and energy saving should be taken into consideration. Based on the above analysis, it may be reasonable to choose the temperature range of 860–900 °C for microwave sintering. 

Secondly, similar to the effect of sintering temperature, the evaluation of relative density, hardness, and flexural strength with the change of cold pressure is performed and the experiment results are shown in Figure 3a and Table 3. It is observed that the value of relative density, hardness, and flexural strength increases by 1.2%, 2.1%, and 4.5%, respectively, with the increase of cold pressure from 150 to 300 MPa, whereas the corresponding indicators increase only 0.2%, 0.3%, and 0.7%, respectively, with the range of cold press from 300 to 450 MPa. It is deduced that the incompact green compacts with large pores are obtained by using small cold pressure, which results in the generated liquid phase is difficult to fill the big pores and finally degenerates the mechanical properties of the sintered samples. Then, as the pressure increases, the decrease of pores in green compact gradually and the increase of diffusion interface facilitate grain-to-grain diffusion and more complete alloying reaction due to the grain boundary moves sufficiently [21,22]. Therefore, the relative density, hardness, and flexural strength are significantly increased. However, the excessive cold pressure causes the decrease in porosity in the compact, which will hinder the penetrability of microwave radiation and decrease sintering efficiency [23]. According to the above analysis, the desired cold pressure should be in the range of 300–450 MPa. 

Thirdly, the holding time has less effect on the relative density, hardness, and flexural strength of the sintered samples. This may ascribe to the unique advantage of microwave heating in the sintering process, that is, the alloying reaction can be completed in a short sintering time and obtain good mechanical properties. This interpretation is verified in Figure 4a and Table 3, and it can be seen that the relative density, hardness, and bending strength are not significantly increased with the increase of the holding time from 15 min to 30 min. However, the continuous increase in holding time will decrease the relative density, hardness, and flexural strength of the samples. The main reason is that the extension of holding time may cause the crystal grains to grow abnormally. At the same time, the elements with a low fusion point will volatilize to generate holes, resulting in the deterioration of relative density, hardness, and flexural strength. Therefore, the desired holding time should be in the range of 30–45 min. 

According to the above comprehensive consideration, the suitable sintering process parameters, such as sintering temperature, cold pressure, and holding time is identified as 860–900 °C, 300–450 MPa, and 30–45 min, respectively. Further, in order to deter mine the appropriate sintering conditions, conditional experiments were carried out on the basis of orthogonal experiments. The results are shown in Figure 2b, Figure 3b and Figure 4b. Looking into the sintering temperature effect (35 min, 375 MPa, 880 to 920 °C), the relative density, hardness, and flexural strength of the sintered samples slightly decrease with temperature increase than decline (Figure 2b). This is as mentioned above, i.e., excessive high sintering temperature lead to grain coarsening and high grain boundary movement rate. When the motion rate of the grain boundary is faster than the rate of gaseous emission, it means that some pores will be surrounded by adjacent grains and resulting in a decline of hardness, relative density, and flexural strength. In addition, we suspect that there is a balance between gas discharge and grain boundary movement. The grain boundary moving rate increases as the sintering temperature increases, but it also increases the probability that the grain boundary prevents gas from being discharged. However, this does not mean that the relative density, hardness, and flexural strength of the samples are reduced, and only when the gas gas discharges is severely hindered. Therefore, the relative density, hardness, and flexural strength of the samples from 880–900 °C are increased. The effect of the cold pressure (35 min, 880 °C) clearly indicates that the relative density, hardness, and flexural strength of the sintered samples slightly decrease with cold pressure increase than decline (Figure 3b). The experimental results are shown in Figure 3b are consistent with those that are analyzed above for Figure 3a. However, when the cold pressure exceeds 450 MPa, the relative density, hardness, and flexural strength of the samples are declined. This may be due to the excessive cold pressure causes the decrease of porosity in the compact, which will hinder the penetrability of microwave radiation and decrease sintering efficiency. The effect of the holding time (375 MPa, 880 °C) reveals that the relative density, hardness, and flexural strength of the samples are in decline with an increase in holding time (Figure 4b). The experimental results are consistent with Figure 4a. In addition, the samples size after sintering was measured as shown in Figure 5. Figure 5 shows that the regular samples can be obtained under the set microwave pressureless sintering conditions. The green compact shrinks to near standard size (30 mm × 12 mm × 6 mm) after microwave pressureless sintering. However, the sintered samples have slightly different sizes under different sintering conditions. It should be noted that the shrinkage process and the densification process of the sample are consistent. Intuitively, denser samples may have better mechanical properties, and the results that are shown in Figure 5 coincide with the results shown in Figure 2b, Figure 3b and Figure 4b.

In summary, when the sintering temperature and cold pressure were increased from 880 to 900 °C, 375 to 450 MPa, the relative density, hardness, and flexural strength of the samples are slightly increased. The relative density, hardness, and flexural strength of the samples are increased when the holding time exceeds 35 min. Based on energy saving considerations, we believe that the MPS conditions chosen should be 880 °C, 375 MPa, 35 min.

**Figure 6:**
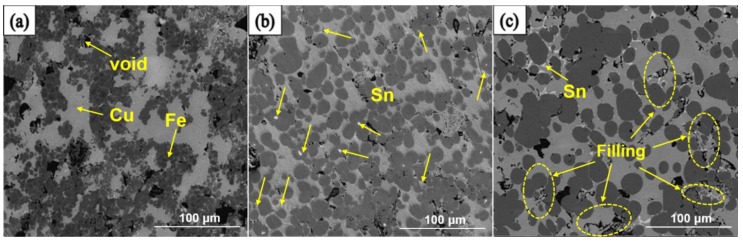
Backscattered scanning electron microscopy (BSE**-**SEM) diagrams of sintered samples by MPS at different sintering temperatures. (**a**–**c**) represent 820 °C, 860 °C and 900 °C, respectively.

**Figure 7:**
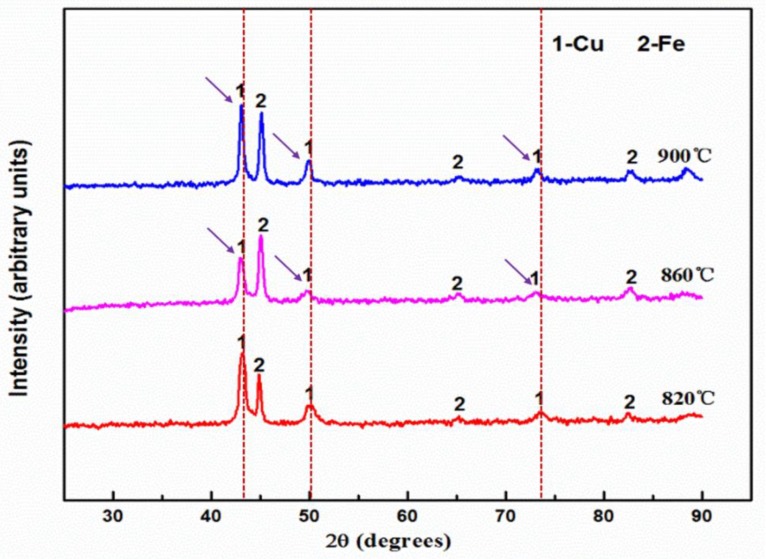
X-ray diffraction (XRD) patterns of samples by microwave sintering at different temperatures.

Based on the above analysis, it can be noticed that the sintering temperature in the MPS method is an uppermost factor to affect the quality of the sintered samples. In order to further reveal the effect of sintering temperature on mechanical properties, the microstructure of samples are tested and the results are shown in Figure 6 and Figure 7. SEM was adopted to confirm the microtopography of sintered Cu-based matrix samples at different sintering temperature and the results were shown in Figure 6. According to our previous work [4], it is confirmed that the region of gray, dark gray, and white in Figure 6 represent Cu-rich phase, Fe-rich phase, and Sn-rich phase, respectively, and the area of dark black stands for the pore. Figure 6a displays the inhomogeneous distribution of the particles when the samples sintered at 820 °C. Many large-sized holes exist and the aggregated behavior of Fe-rich and Cu-rich phase can be observed. With the increase of sintering temperature up to 860 °C, as shown in Figure 6b, the amount of voids is reduced and the Fe-rich phase in the alloy matrix diffuses into the Cu-rich phase to achieve an overall uniform distribution. This may be derived from a fact that the excellent migration efficiency of metallic elements and liquid phase rearrangement behavior in the matrix with the increase of sintering temperature. As the sintering temperature increases up to 900 °C, as manifested in Figure 6c, it is observed that the evident growth of pores and grains. This is mainly because the formation of adequate amounts of the liquid phase at high temperature facilitates the continuous dissolution of metallic powder particles, which leads to recrystallization and abnormal grain growth. Furthermore, the emergence of holes results from the loss of the low melting point element at high temperature. It is worth mentioning that the evolution regularity of microstructure with the change of sintering temperature can account for the results of mechanism performance in Table 4 and Figure 2. 

In order to investigate the migration behavior of main metallic elements with the sintering temperature, XRD test of sample sintered at different temperatures was performed and the results were shown in Figure 7. It is observed that the slightly blue shift for the characteristic diffraction peak of Cu with the increase of sintering temperature, meanwhile the intensity of the Cu characteristic peak rises after an initial decline. The strong intensity of the diffraction peak at 820 °C may attribute to the accumulation of copper and the incorporation of other elements in the copper lattice at low sintering temperatures [24,25]. With the increase of sintering temperature up to 860 °C, the appearances of blue shift and intensity reduction of the Cu diffraction peak should be ascribed to liquid phase Sn elements enter Cu lattice and then cause lattice distortion. As to the rising tendency of Cu diffraction peak intensity at 900 °C, it is indicating that the copper component diffuses and accumulates at high temperatures, which is also related to the evolution of microstructure in Figure 6.

### 3.2. Comparison of Microwave and Conventional Sintering

**Figure 8:**
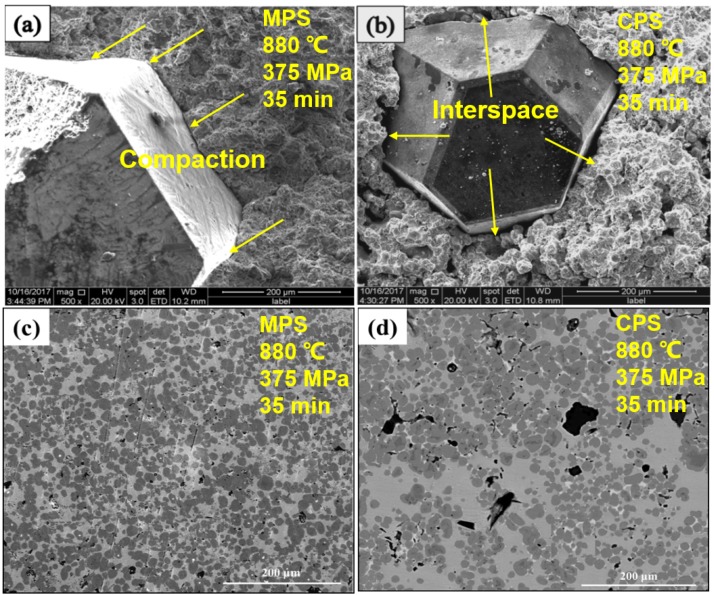
Backscattered scanning electron microscopy (BSE-SEM) pictures of microstructure morphology of samples sintered by microwave (**a**,**c**) and conventional sintering (**b**,**d**).

**Figure 9:**
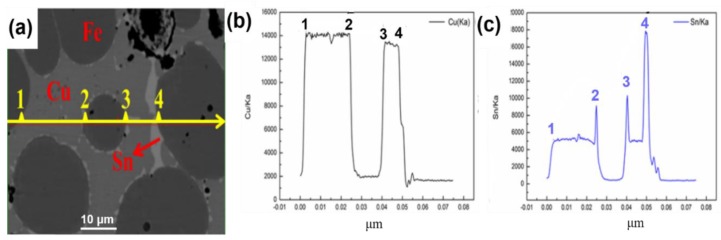
EPMA line scan analysis of the sample by microwave sintered at 880 °C: (**a**) EPMA image, (**b**) Cu content change, (**c**) Sn content change.

**Figure 10:**
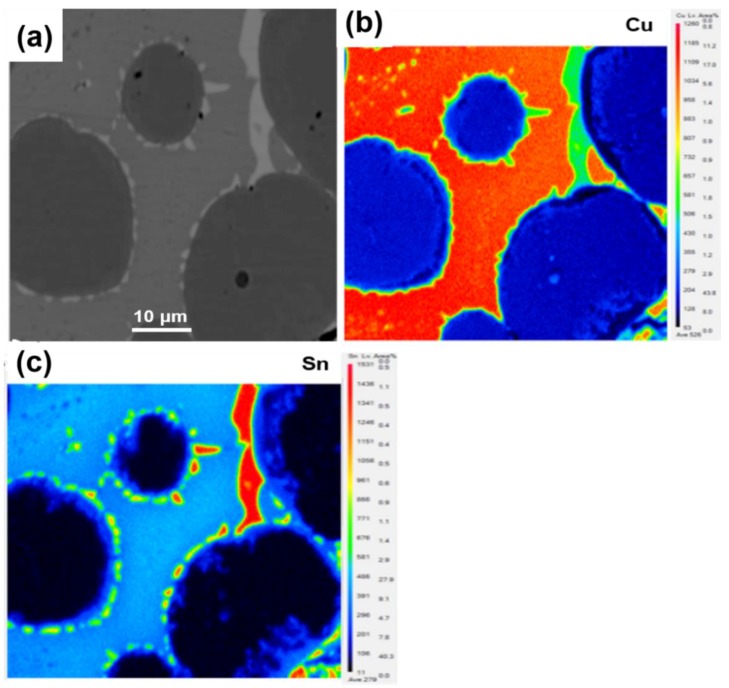
Electron probe microanalysis (EPMA) mapping scan analysis of the sample by microwave sintered at 880 °C: (**a**) EPMA image, (**b**) distribution of Cu, (**c**) distribution of Sn.

To further contrast the discrepancy of sintering method by microwave and conventional heating, the same process condition, i.e., the optimum MPS process parameter 880 °C/375 MPa/35 min, is selected for MPS and CPS, respectively. In addition, an attempt to prepare the diamond-containing (mass percentage, 3%) Cu-based metal matrix by MPS and CPS is also performed under the above sintering process condition, and the experimental results are shown in Table 4 and Figure 8, Figure 9 and Figure 10. From Table 4, the average relative density of microwave sintered samples is 94.12%, which is 1.23% higher than that of conventional sintering. It shows a better densification degree of microwave sintered samples under the same process conditions. At the same time, compared to conventional sintering, the hardness, flexural strength-N (no addition of diamond abrasive), flexural strength-C (containing diamond abrasive) and holding force coefficient of microwave sintered samples also increased by 3.72%, 5.91%, 9.99%, and 4.35%, respectively. Figure 8a,b are the fracture morphology of the sample after MPS and CPS. It can be clearly seen that the sample has fewer fracture pores by MPS, and the sample after CPS has more holes. At the same time, it can be found that the diamond abrasives tightly bound to the metal matrix by the MPS method, whereas there is a large gap between the diamond and matrix by the CPS method, which means that the holding force coefficient of samples by microwave sintering is obviously more excellent than that of conventional sintering. From Figure 8c,d, the samples that are sintered by MPS have more uniform microstructure, smaller porosity, and fine crystal grain size. In summary, the mechanical properties of sintered samples that were prepared by the MPS method are significantly better than the CPS method. For this reason, we attempt to explain these phenomena from element diffusion and alloying reactions and EPMA spectra results of samples sintered by MPS are shown in Figure 9 and Figure 10. As manifested in Figure 9a, the light gray area represents Cu-rich phase, the dark gray represents Fe-rich phase, the red arrow indicates Sn-rich phase, and the yellow arrow indicates the direction of line scanning. Figure 9b,c are the changes of the Cu and Sn elements in the substrate along the sweep direction, respectively. It can be observed that the content of Cu and Sn increases rapidly in the scan region from point 1 to point 2. The signal of Cu and Sn disappear when the scan region from point 2 to point 3. It is worth mentioning that strong the signal intensity of Sn emerged in point 2, 3, and 4, which means that the Sn element accumulates around the Fe-rich phase as a liquid filling phase in the process of sintering. As a consequence, in the area of Cu-rich phase, there is a similar change trend for Cu and Sn elements, which indicates the sufficient alloying reaction of Cu and Sn to produce the Cu-Sn alloy or solid solution phase. In addition, Sn phase is enriched at the binding boundary between Fe-rich phase and Cu-rich phase to act as a binder to improve the mechanical properties of the metallic matrix. To further investigate the situation of metallic element distribution, EPMA mapping analysis is adopted and the results are shown in Figure 10. As figuratively displayed in Figure 10b,c, the distribution of Cu and Sn elements in the matrix is obtained, and the color depth in the graph indicates the concentration of corresponding elemental distribution. It is found that the Sn elements aggregate in the interface of Cu-Fe, while it uniformly distributes in the Cu-rich phase. The main reason is that Cu and Sn generate a part of a Cu-Sn liquid phase in the sintered body, and the Cu-Sn liquid phase first wets the surface of Fe skeleton at a sintering temperature of 880 °C [18,26]. In addition, the inefficient diffusion rate of Cu-Sn liquid phase in the iron skeleton and high diffusion rate of Sn to Fe-rich phase, which results in the diffusion of Sn liquid phase into the sintering necks between Fe and Cu grains. Therefore, abundant Sn phase distributes around the neck. It is precisely due to the diffusion of Cu-Sn liquid phase that the pores are continuously filled, meanwhile, the mechanical properties of the sintered sample are improved due to the reinforcing effect of Cu-Sn alloy between the different particles.

### 3.3. The Possible Mechanism of Microwave Pressureless Sintering

**Figure 11:**
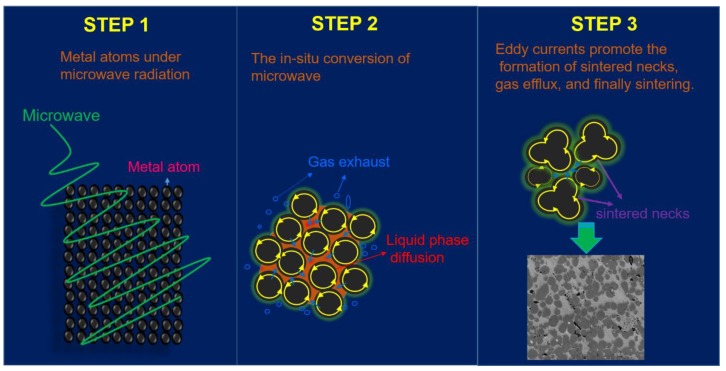
The schematic diagram of a possible mechanism for the microwave pressureless sintering.

According to the above discussion, the Cu-based metal matrix was successfully prepared by MPS and the quality of samples that were sintered by MPS and CPS methods were comparatively evaluated. It was found that the mechanical properties of samples sintered by the MPS method were significantly better than those of the CPS method. The diversity of mechanical properties may stem from the different heating principles of microwave and conventional sintering. In the absence of pressure, for conventional sintering processes, the metal block absorbs thermal energy through the convection and conduction modes of the heat source, usually some high resistance heating elements [9]. Densification of sintered samples is accomplished by the diffusion of liquid phase elements and alloying reactions. However, the temperature gradient from the outside to the inside that is generated by the conventional heating method may hinder the discharge of gas inside the sintered body [13,17]. Obviously, this is very detrimental to the diffusion of elements, and thus affects the quality of sintered samples. Unlike conventional heating, the microwaves start heating from the inside of the material, and the resulting temperature gradient is consistent with the direction of gas discharge, which facilitates the densification of the sintered sample and the formation of a uniform microstructure. In addition, metal atoms may directly absorb microwave energy and be converted to activate sintering, which facilitates alloying reactions. This is consistent with the analysis of Figure 5, Figure 6, Figure 7, Figure 8 and Figure 9. Therefore, we proposed the possible MPS mechanism that is shown in Figure 11. It is generally believed that the microwave heating of metals can be simply divided into two processes, the formation of the sintering neck before and after [27,28,29]. During the early stage of MPS, the green compactors have a certain void that can receive microwave radiation and be heated. The microwave energy is absorbed by metal powders and the behavior of in situ energy conversion will increase the activity of metal atoms, in turn, facilitate the diffusion of elements and alloying reactions [24,30]. In addition, the magnetic permeability loss mechanism of metal particles may also have a similar effect [9]. The appearance of sintering neck is accompanied by the formation of a liquid phase, and the liquid phase element diffuses to fill the internal pores. For diamond-containing sintered samples, the liquid phase elements also bond tightly with the diamond to enhance the diamond holding ability in the matrix. As the previous analysis, the temperature is the main factor in the process of sintering and high temperatures can promote the formation of liquid phase elements. Demirskyi et al. found that the liquid phase appeared around tungsten carbide sintering neck at the sintering temperature of 950 °C in the microwave field in spite of the temperature were far lower than the melting temperature of WC [27]. It means that microwave sintering can form a local liquid phase at low temperature, and then the diffusion and migration of liquid phase elements will promote the holding force of diamond abrasives in metallic matrix due to the filling effect. This may be one reason to account for the results of Figure 8. In addition, SiC assisted heating is required to provide thermal energy with the gradual densification of green compacts because of the weak penetrability of microwave radiation in quasi-bulk metal. 

Through the above analysis and our previous work, we have reason to believe that the unique heating mode of microwaves is conducive to the rapid shrinkage of microstructures and the reduction of porous defects to enhance the mechanical properties of the sintered samples.

## 4. Conclusions

Cu based metallic binder for diamond tools was successfully prepared via microwave pressureless sintering method by adopting single metal powders as raw materials. The orthogonal design is used to optimize the sintering process conditions, and the diamond-containing metal matrix was prepared under optimized reaction conditions by the MPS and CPS methods, respectively. It is reasonable to speculate that MPS shows potentiality promising application in the manufacture of diamond tools through the study of the MPS method for the preparation of metal matrix for diamond tools. The main findings are summarized, as follows:

(1) MPS temperature is the main factor affecting the quality of sintered samples. Increasing the temperature is beneficial to the improvement of the mechanical properties of the Cu based metal matrix during the microwave pressureless sintering process. However, excessively high sintering temperature will lead to grain growth in the metal matrix, which is not conducive to the improvement of mechanical properties.
(2) Comparative analysis of the samples that were sintered by MPS method and CPS method under the suitable sintering conditions (880 °C, 375 MPa, 35 min), it was found that the MPS is advantageous to improve the mechanical properties of the metal matrix and to promote the holding force of diamond abrasives in a metallic matrix.
(3) Before the sintering neck is formed, the in situ conversion energy mode can enhance the diffusion and alloying process when the green compacts are exposed to microwave irradiation. The phase distribution of the MPS sample is more uniform than the CPS sample, as a prerequisite for the same ingredient and sintering conditions.

This work is a successful attempt to provide a possibility to expand the microwave thermal energy application into the sintering field for metallic binder diamond tools. It also provides a reliable reference for the promotion of MPS preparation of metal-based diamond tools. However, further investigation should be taken for the application of MPS technology in metallic matrix, such as more systematic sintering mechanisms and more energy efficient process.

## Figures and Tables

**Table 1:** Composition of metallic-based bond.

Ingredient	Cu	Fe	Co	Sn	Ni	Ti
Content (wt %)	40	30	13	7	8	2

**Table 2:** The results of the orthogonal experiment.

Experiment Number	Factors			Indexes		
	A: Sintering Temperature (°C)	B: Cold Pressure (MPa)	C: Holding Time (min)	Relative Density (%)	Hardness (HRB)	Flexural Strength (MPa)
1	820	150	15	85.55	81.2	526
2	820	300	30	87.83	85.7	596
3	820	450	45	87.90	84.5	590
4	860	150	30	92.49	91.7	732
5	860	300	45	92.73	92.6	749
6	860	450	15	93.62	93.9	760
7	900	150	45	93.13	94.8	750
8	900	300	15	93.86	95.1	758
9	900	450	30	93.51	95.8	768

**Table 3:** The results of orthogonal test range analysis.

Mechanical Properties	Project	Factors		
		Sintering Temperature (°C)	Cold Pressure (MPa)	Holding Time (min)
cRelative density (%)	*X_e_*_1_	261.28	271.17	273.03
	*X_e_*_2_	278.84	274.42	273.83
	*X_e_*_3_	280.50	275.03	273.76
	*X_e_*_1_/3	87.09	90.39	91.01
	*X_e_*_2_/3	92.95	91.47	91.28
	*X_e_*_3_/3	93.50	91.68	91.25
	R (range)	6.41	1.29	0.27
	Rank	1	2	3
Hardness (HRB)	*Y_e_*_1_	251.40	267.70	270.20
	*Y_e_*_2_	278.20	273.40	273.20
	*Y_e_*_3_	285.70	274.20	271.90
	*Y_e_*_1_/3	83.80	89.23	90.07
	*Y_e_*_2_/3	92.73	91.13	91.07
	*Y_e_*_3_/3	95.23	91.40	90.63
	R (range)	11.43	2.17	1.00
	Rank	1	2	3
Flexural strength (MPa)	*Z_e_*_1_	1712.00	2008.00	2044.00
	*Z_e_*_2_	2241.00	2103.00	2096.00
	*Z_e_*_3_	2276.00	2118.00	2089.00
	*Z_e_*_1_/3	570.67	669.33	681.33
	*Z_e_*_2_/3	747.00	701.00	698.67
	*Z_e_*_3_/3	758.67	706.00	696.33
	R (range)	188.00	36.67	17.34
	Rank	1	2	3

**Table 4:** Performance comparison between microwave and conventional sintered samples.

Method	Relative Density (%)	Hardness (HRB)	Flexural Strength (MPa)		Holding Force Coefficient (%)
			No Diamond	Containing Diamond	
MPS	94.12	95.1	766	664	86.68%
	94.13	95.3	768	667	86.85%
	94.11	95.4	769	670	87.13%
**Average**	94.12	95.27	767.67	667.00	86.89%
CPS	92.90	91.7	720	597	82.92%
	92.87	91.6	722	600	83.10%
	93.10	91.9	725	604	83.31%
**Average**	92.96	91.73	722.33	600.33	83.11%
